# Unraveling Genetic Diversity and Antibiotic Resistance Mechanisms in *Citrobacter freundii* Isolates From Meat Products: Implications for Food Safety and Public Health

**DOI:** 10.1002/vms3.70493

**Published:** 2025-10-22

**Authors:** Hussein Khodabandeh, Elaheh Tajbakhsh, Faham Khamesipour, Hassan Momtaz, Manochehr Momeni shahraki

**Affiliations:** ^1^ Department of Microbiology Faculty of Basic Sciences Shahrekord Branch Islamic Azad University Shahrekord Iran; ^2^ Food and Drug Research Center Iran Food and Drug Administration, Ministry of Health and Medical Education Tehran Iran; ^3^ Department of Food Hygiene and Quality Control Faculty of Veterinary Medicine Shahrekord Branch Islamic Azad University Shahrekord Iran

**Keywords:** biofilms, *Citrobacter freundii*, drug resistance, food contamination, microbial, meat products, polymerase chain reaction, zoonoses

## Abstract

*Citrobacter freundii* is a zoonotic bacterium with special public health importance as a carrier and source of multidrug resistance (MDR) genes. Here, the prevalence, genetic heterogeneity, and antimicrobial resistance pattern of *C. freundii* from chicken and red meat in Shahrekord, Iran, were examined. Cross‐sectional design and a combination of molecular methods with random amplified polymorphic DNA (RAPD‐PCR), enterobacterial repetitive intergenic consensus (ERIC‐PCR), and repetitive extragenic palindromic PCR (REP‐PCR) and phenotypic methods were used for identifying genotypic patterns and resistance markers such as *bla_CTX‐M_*, q_nr_ (within PMQR plasmids), and sul1 genes. RAPD‐PCR was the most discriminative and identified significant genetic diversity in the poultry isolates. The findings describe an alarming prevalence of MDR with resistance patterns correlated with farming practice and antibiotic exposure. Seasonal patterns in the degree of contamination were also described, highlighting focused intervention and judicious antimicrobial stewardship. The research emphasises the utilisation of integrated surveillance systems and molecular diagnostic approaches to mitigate the public health issues resulting from *C. freundii* throughout the food chain.

## Graphical abstract

This study highlights the high prevalence and multidrug resistance of *Citrobacter freundii* in poultry meat, revealing diverse genotypes and key resistance mechanisms through integrated molecular diagnostics and clustering analysis.

A graphical abstract summarising the main findings and methodology of this study is provided as a separate file (Graphical_Abstract.png). These visuals highlight the prevalence of *Citrobacter freundii*, its multidrug resistance mechanisms, and the molecular diagnostic approaches applied to assess genetic diversity and antimicrobial resistance patterns in meat products.

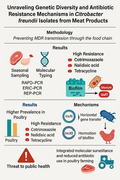

## 1 Introduction

*Citrobacter freundii* presents a significant food safety and public health issue because of its dual role as an opportunistic pathogen and zoonotic pathogen. The facultative anaerobic, Gram‐negative has been gaining notoriety as a causative agent of nosocomial infections and outbreaks from foodborne disease, as it has effectively elevated environmental reservoirs and clinical settings via the spread of antimicrobial resistance (AMR) determinants (Johnson et al., 2020; Pletz et al. 2018). It should be noted *C. freundii* has repeatedly been associated with food vehicles like red meat, poultry, fish, and ready‐to‐use ones on a regular basis, with many primary critical control elements of the food chain interacting with microbial contamination and AMR (Anas et al. 2019; Uzeh et al. 2021; Al‐Haider et al. 2019; Sheng and Wang 2021). The ubiquitous presence of foodstuffs represents a considerable risk to human health, accelerates multidrug‐resistance dissemination globally, and jeopardises food security (Mohammed & Al‐Samarraae, 2021).

The rise of AMR across the globe has been recognised as one of the most significant public health threats of the 21st century that has largely been instigated through the overuse and misuse of antimicrobials in treating human disease and in agriculture (Sheng and Wang 2021). Bacteria such as *C. freundii* are the crux of the problem, as it represents a vast reservoir of MDR organisms, subsets of which influence horizontal gene transfer between species and within genera. The non‐limiting broad‐spectrum antibiotic resistance determinants of greatest concern are related to the resistance factors to extended‐spectrum cephalosporins, β‐lactams, and sulphonamides that include *bla_CTX‐M_, bla_TEM_, bla_NDM_*, and *sul1*, respectively (Meini et al., 2019; Bunyan 2020; Mahmood and Atyah 2021). These patterns of resistance exist in self‐replicating configurations such as plasmids, transposons, and integrons, promoting *C. freundii's* adaptability and environmental persistence through a wide range of niches (Pletz et al. 2018; Liu et al. 2018).

Epidemiologically significant information regarding *C. freundii* genetic heterogeneity and AMR mechanisms is important to develop effective intervention programmes and surveillance programs. Molecular typing reagents such as Random Amplified Polymorphic DNA (RAPD‐PCR), Enterobacterial Repetitive Intergenic Consensus (ERIC‐PCR), and Repetitive Extragenic Palindromic PCR (REP‐PCR) provide information of great value concerning the genetic makeup and population structure of the pathogen. Of those, RAPD‐PCR, which has resolution so high that it is useful for differentiating closely related strains, and REP‐PCR, which has a reproducibility‐resolution ratio suitable for following longitudinal studies, are both of great utility for differentiating large genetic clusters, providing a macro‐level perspective (Bunyan 2020; Al‐Daraghi and Al‐Behadili 2020; Uzeh et al. 2021). They have been demonstrated to be useful; however, comparative molecular typing methods are not common in typing *C. freundii* foodstuffs, isolates, and therefore there is a need for more.

The high‐quality molecular tests for *bla_CTX‐M_*, *bla_TEM_*, and *sul1* have identified molecular determinants that are the primary vectors facilitating the transmission of antimicrobial resistance (AMR) between bacterial populations. The *bla_CTX‐M_* gene coding for extended‐spectrum *β*‐lactamases (ESBLs) is listed currently as one of the most disseminated AMR markers the world over, and it has a significant impact on third‐generation cephalosporins, as it works through ESBLs that undermine its effectiveness in veterinary and human medicine (Bunyan 2020). Similarly, the *bla_TEM_* determinant confers resistance to *β*‐lactam antibiotics such as derivatives of penicillin, whilst *sul1* confers sulfonamide agents once considered an integral component of farm antimicrobial regimens (Mahmood and Atyah 2021). The ability of *C. freundii* to harbour these genes indicates its remarkable flexibility and helps to underscore the importance of horizontal gene transfer in the rapidity of resistance of its transmission (Uzeh et al. 2021).

Although molecular typing techniques such as RAPD‐PCR, ERIC‐PCR and REP‐PCR have been a suitable avenue for genotyping and characterisation of AMR, more recent studies indicate that these molecular findings must also be analysed with the resistance phenotypes' data in order to better explore the epidemiological complexity of MDR pathogens (Al‐Daraghi and AL‐Behadili 2020). For example, RAPD‐PCR has proved useful in tracking point sources of contamination in foodborne outbreaks, and useful information was gained from it regarding an outbreak's eventual control and management (Bunyan 2020). REP‐PCR has been employed to establish clonal relationships of *C. freundii* isolates from distinct environmental niches and illustrate clonal relationships and pathways of potential dissemination (Uzeh et al. 2021). All of these findings bring to light that molecular methods are absolutely essential not only to characterise AMR markers but also tracking the evolutionary processes of resistance dissemination.

The public health ramifications of these genetic findings are much larger than academic research implications and are immediately relevant to infection control strategies and food safety policy. For instance, the repeated isolation of *bla_CTX‐M_* and *sul1* from both poultry and red meat product isolates indicates an immediate risk for greater restriction of on‐farm antibiotic use (Sheng and Wang 2021). *C. freundii* provides evidence of a specific food‐chain transmissible vehicle for antimicrobial resistance (AMR), and it presents a real level of risk to the therapeutic value in the clinic and puts infection control strategies to their limits (Pletz et al. 2018). Such risks call for more genomic surveillance and encourage responsible antimicrobial use to protect public health and the global food supply stability.

To this end, the current paper answers some of the greater knowledge gaps, using the assessment of the prevalence, genetic variation, and antimicrobial resistance patterns of *C. freundii* strains isolated from poultry and red meats in Shahrekord, Iran. By using an integrated approach using sophisticated molecular techniques (RAPD‐PCR, ERIC‐PCR, REP‐PCR) with a significant amount of phenotypic antimicrobial resistance testing, the current study aimed to identify the molecular and phenotypic antimicrobial resistances and spend time classifying them based on their potential for contamination that varied with seasons. Using genomic and phenotypic data, this study gives a complete picture of *C. freundii* as a zoonotic pathogen and contributing bacterium to the global AMR disease pandemic we are currently experiencing. Ultimately, these findings will inform integrated food safety and public health solutions to make actionable progress toward addressing the risks surrounding the spread of AMR pathogens through the food supply chain.

## 2 Methodology

### 2.1 Study Design and Population

This study employed a cross‐sectional descriptive design to evaluate the prevalence, antibiotic resistance, biofilm formation, and genetic diversity of *C. freundii* isolates from meat samples.

- **Study population**: The study focused on red meat (beef, mutton, veal) and poultry (chicken, quail, turkey) samples collected from retail outlets in Shahrekord, Iran.
- **Sampling period**: Samples were collected seasonally across the four seasons of 2023 (spring, summer, autumn, and winter) to identify potential seasonal variations in contamination rates.
- **Sampling location**: All samples were sourced from randomly selected retail outlets in Shahrekord, ensuring a representative selection of the region's meat supply chain.
- **Sample size determination**: The sample size was calculated as 600 using Cochran's formula: n = $\frac{z^{2}\;.\;p\;.\;q}{d^{2}}$
- where:
  - *z* = 1.96, the standard normal deviation for a 95% confidence level.
  - *p* = 0.3 Estimated prevalence of *C. freundii* from prior studies.
  - *q* = 1− *p* = 0.7 Complementary probability.
  - *d* = 0.05 Desired margin of error.

This formula ensured sufficient statistical power to detect meaningful differences in prevalence and characteristics across the isolates studied (Barbour et al. 2018; Kumar et al. 2023).

### 2.2 Microbial Isolation and Identification

#### 2.2.1 Sample Preparation

To prepare the samples for microbial analysis, 10 grams of each meat sample were homogenised in 90 millilitres of Tryptic Soy Broth (TSB). This process was performed using a stomacher to ensure consistent mixing of the samples. The homogenised mixtures were then incubated at 37 degrees Celsius for 24 h to enrich the bacterial population, maximising the likelihood of detecting *C. freundii* (ISO, 2017; Nossair et al., 2015).

#### 2.2.2 Selective Culturing

For selective isolation of *C. freundii*, the enriched broth was plated on Xylose Lysine Deoxycholate (XLD) agar and Eosin Methylene Blue (EMB) agar. Both media were procured from Merck, Germany. The inoculated plates were incubated at 37 degrees Celsius for 24 h. Presumptive colonies of *C. freundii* were identified based on characteristic growth patterns: pink‐coloured colonies on MacConkey agar and a metallic sheen on EMB agar, both indicative of lactose fermentation (Al‐Iedani et al., 2015).

#### 2.2.3 Biochemical Confirmation

Biochemical tests were conducted to confirm the identity of presumptive *C. freundii* isolates. The following tests were performed:

- Gram staining: To determine the Gram reaction and morphology of the isolates.
- Simmon's citrate test: To assess the utilisation of citrate as a sole carbon source.
- Indole production test: To determine the production of indole from tryptophan.
- Methyl red and Voges‐Proskauer tests: To evaluate the metabolic pathways used in glucose fermentation.
- Hydrogen sulphide production test: To detect the generation of hydrogen sulfide gas.
- Motility test: To assess bacterial motility.

The standard strain *C. freundii* ATCC 13316 was used as a positive control in all identification steps to ensure accuracy and consistency (Ashish et al. 2023; Rasheed 2012).

### 2.3 Antibiotic Susceptibility Testing

Antibiotic susceptibility testing was performed using the Kirby‐Bauer disc diffusion method in accordance with Clinical Laboratory Standards Institute (CLSI) guidelines (Wayne, 2019). Bacterial suspensions were standardised to a turbidity equivalent to a 0.5 McFarland standard. This was achieved by preparing bacterial suspensions in sterile saline and adjusting their optical density using a spectrophotometer.

Inoculated Mueller‐Hinton agar plates were used for testing, and antibiotic discs for ampicillin, cefotaxime, erythromycin, gentamicin, and ciprofloxacin were applied. Plates were incubated at 37 degrees Celsius for 24 h, after which inhibition zones were measured and classified as resistant, intermediate, or susceptible according to CLSI standards (Begum et al. 2019; Uzeh et al. 2021).

### 2.4 Biofilm Formation Assay

The biofilm‐forming ability of isolates was assessed using the microtiter plate method as described by Yang et al. (2021). Standardised bacterial suspensions were inoculated into 96‐well plates containing TSB supplemented with one percent glucose. The plates were incubated at 37 degrees Celsius for 48 h to allow biofilm formation. After incubation:

1. Wells were washed three times with phosphate‐buffered saline (PBS) to remove non‐adherent cells.
2. Biofilms were fixed with methanol for 15 min and stained with 0.1 percent crystal violet for 15 min.
3. Excess dye was removed, and the wells were washed again with PBS.

The biofilm's intensity was quantified by measuring the optical density at 570 nanometres using a spectrophotometer. Biofilm formation was categorised as weak, moderate, or strong based on the optical density values compared to control wells (Al‐Daraghi and AL‐Behadili 2020; Yang et al. 2021).

### 2.5 Detection of Antibiotic Resistance Genes

The presence of antibiotic resistance genes, including *bla_CTX‐M_*_,_ *bla_TEM_*, *bla_SHV_*, *sul1*, *qnrA*, *qnrB*, and *tetA*, was detected using Polymerase Chain Reaction (PCR). The primers used for amplification were designed based on published sequences (Jia et al. 2020; Praharaj et al. 2016) and validated through BLAST analysis against the NCBI database (Hassen et al. 2020; Jia et al. 2020).

The PCR mixture consisted of:

- 2.5 microlitres of 10X PCR buffer,
- 0.5 microlitresof 10 millimolar dNTPs,
- 0.75 of 50 millimolar magnesium chloride,
- 1 microlitre each of forward and reverse primers (10 micromolar),
- 0.5 of Taq DNA polymerase (5 units per microlitre),
- 1 of DNA template (∼50 nanograms per microlitre), and
- Sterile distilled water to a total reaction volume of 25 microlitres.

Thermal cycling conditions were as follows:

1. Initial denaturation at 95 degrees Celsius for 5 min.
2. Thirty cycles of:
  - Denaturation at 95 degrees Celsius for 60 s,
  - Annealing at temperatures ranging from 56 to 60 degrees Celsius for 60 s, and
  - Extension at 72 degrees Celsius for 90 s.
3. Final extension at 72 degrees Celsius for 7 min.

### 2.6 Genotyping and Molecular Typing

#### 2.6.1 ERIC‐PCR

Enterobacterial Repetitive Intergenic Consensus (ERIC)‐PCR was performed using primers ERIC‐1 and ERIC‐2. The PCR conditions followed the optimised protocol by Ranjbar et al. (2017). This method was used to evaluate the genetic diversity among isolates (Bughti et al. 2017; Singh et al. 2024).

#### 2.6.2 RAPD‐PCR

Random Amplified Polymorphic DNA (RAPD)‐PCR utilised a 10‐nucleotide primer with the sequence GCATCCCCA, as described by Vedovik et al. (2006). PCR conditions included initial denaturation at 94 degrees Celsius for 4 min, followed by 30 cycles of denaturation, annealing, and extension, with a final extension at 72 degrees Celsius for 5 min (Adinortey et al. 2020; Zhang et al. 2024).

#### 2.6.3 REP‐PCR

Repetitive Extragenic Palindromic (REP)‐PCR used primers REP‐1 and REP‐2 under the conditions described by Mechlim et al. (2008). This technique further validated the genotypic relationships among the isolates (Pan et al. 2021; Ullah et al. 2024a).

### 2.7 Gel Electrophoresis

PCR products were analysed using 2 percent agarose gel electrophoresis. The gels were stained with DNA Safe Stain (CinnaGen, Iran) and run in 1X TBE buffer at 90 volts for 60 min. DNA bands were visualised under UV light using a gel documentation system (Al‐Musawi et al. 2021; O'Hara and Miller 2003).

### 2.8 Statistical Analysis

All data were analysed using SPSS version 22.0. Statistical significance was determined using chi‐square tests and ANOVA, with a *p*‐value of less than or equal to 0.05 considered significant. Molecular typing profiles were analysed using GelJ software, which generated dendrograms based on the Dice coefficient and UPGMA clustering (SAS, 2012; Barbour et al., 2018).

## 3 Results

### 3.1 Prevalence of *C. freundii* in Various Meat Types

This section presents the prevalence of *C. freundii*, a gram‐negative bacterium commonly associated with foodborne illnesses, in different types of meat. Table 1 summarises the total number of samples tested, the number of positive samples, and the corresponding prevalence percentage. The data highlights a higher prevalence in poultry products, with quail showing the highest rate (12%). Among red meats, lamb exhibited the highest prevalence (9%).

**TABLE 1:** Prevalence of *C. freundii* in different meat types.

Sample type	Total samples	Positive samples	Prevalence (%)
Beef	100	2	2%
Veal	100	4	4%
Lamb	100	9	9%
Total Red Meat	300	15	5%
Chicken	100	7	7%
Quail	100	12	12%
Turkey	100	5	5%
Total Poultry	300	24	8%

Among red meats, lamb had the highest prevalence (9%), whereas beef and veal showed relatively lower rates (2% and 4%, respectively). In total, 300 red meat samples were analysed, yielding an overall prevalence of 5%. In poultry, *C. freundii* was more prevalent, particularly in quail (12%), followed by chicken (7%) and turkey (5%). The overall prevalence in poultry samples was 8%.

These findings underscore the varying contamination levels of *C. freundii* in different meat types, with poultry showing higher rates than red meat. This highlights the critical need for enhanced food safety measures in the poultry industry to minimise public health risks.

### 3.2 Antibiotic Resistance Profiles of *C. freundii* Isolates

**FIGURE 1:**
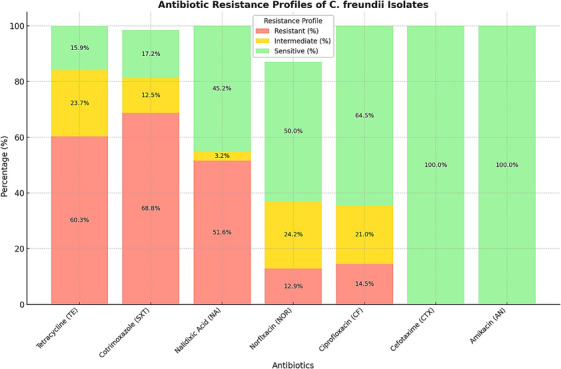
Antibiotic resistance profiles of *C. freundii* isolates from red meat and poultry samples. This figure displays resistance, intermediate sensitivity and sensitivity percentages for *C. freundii* isolates against seven commonly used antibiotics in human and veterinary medicine.

The analysis of antibiotic resistance profiles in *C. freundii* isolates, depicted in Figure 1, reveals alarming trends in antimicrobial resistance. The data indicate high resistance rates to widely used antibiotics, particularly tetracycline (60.3%), cotrimoxazole (68.8%), and nalidixic acid (51.6%), suggesting a diminishing efficacy of these drugs in treating *C. freundii* infections. Notably, resistance was more pronounced in poultry isolates, likely due to unregulated antibiotic use in intensive farming practices.

Moderate resistance levels were observed for norfloxacin (12.9%) and ciprofloxacin (14.5%), accompanied by notable intermediate sensitivity rates (24.2% and 21.0%, respectively). These trends suggest a partial reduction in efficacy for these antibiotics, necessitating careful monitoring. On a positive note, all isolates were completely sensitive to cefotaxime and amikacin, positioning these antibiotics as effective treatment options for *C. freundii* infections.

The findings underscore the urgent need for antibiotic stewardship programmes, especially in the poultry sector, where overuse of antibiotics is prevalent. Surveillance programmes should monitor resistance trends to prevent the emergence of multidrug‐resistant (MDR) strains.

These results have significant implications for public health and food safety. Addressing the widespread resistance to commonly used antibiotics is critical to mitigate risks. The continued efficacy of cefotaxime and amikacin provides hope for effective therapeutic options; however, judicious use is essential to preserve their efficacy. Public education on safe meat handling, combined with robust regulatory frameworks, can further reduce the risks associated with antibiotic‐resistant *C. freundii*.

### 3.3 Biofilm Formation and Antibiotic Resistance in *C. freundii*

**FIGURE 2:**
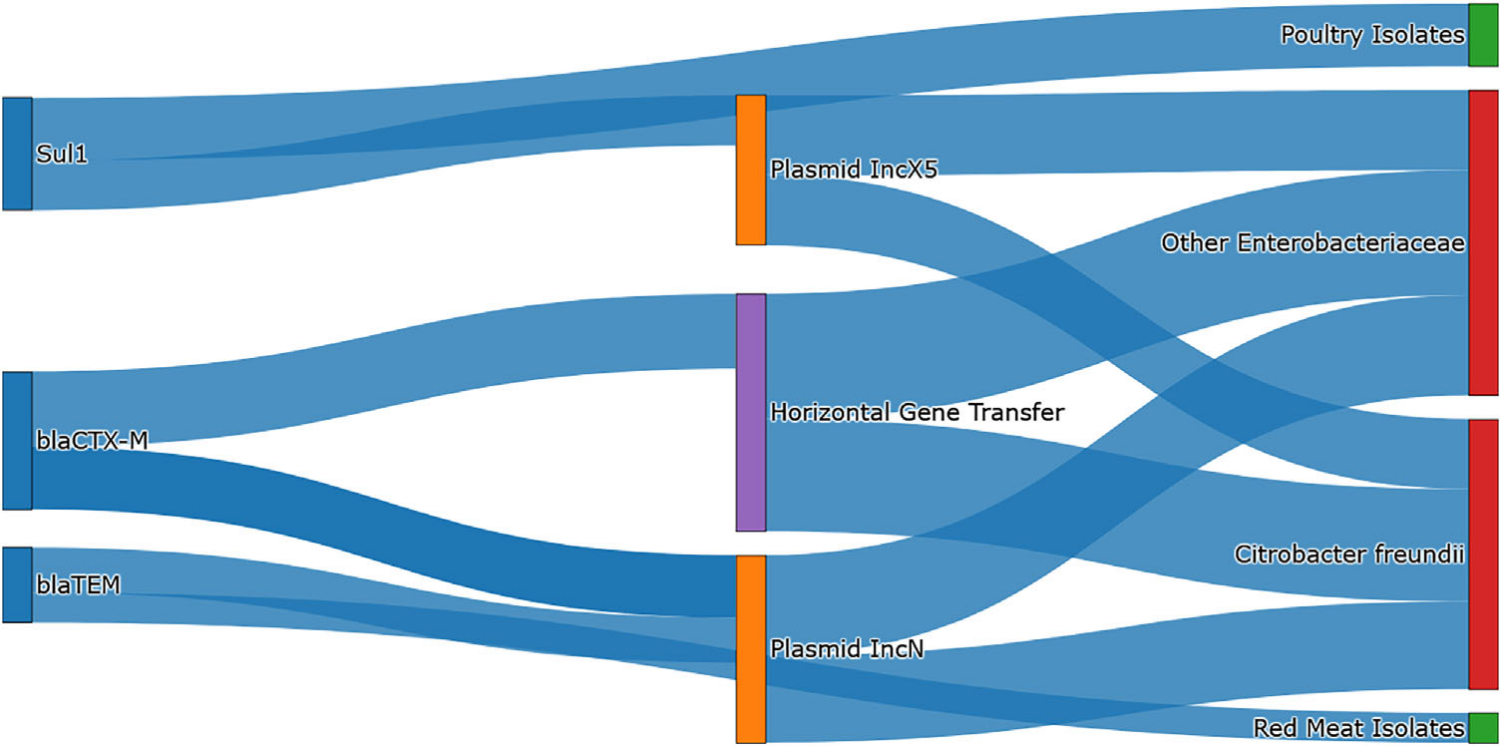
Genetic flow and dissemination of resistance genes in *C. freundii* isolates from food sources.

The results presented in Figure 2 highlight the significant role of biofilm formation in enhancing the persistence and resistance of *C. freundii* in various environments, including clinical and food processing systems. The majority of *C. freundii* isolates (61.5%) demonstrated strong biofilm formation capabilities, which is a critical factor in increasing antibiotic resistance and survival in challenging conditions. Moderate biofilm formation was observed in 38.5% of the isolates, further underscoring the adaptive resilience of this bacterium.

#### 3.3.1 Biofilm Formation and Antibiotic Resistance

The data reveals a significant association between strong biofilm formation and resistance to key antibiotics, including tetracycline (33.3%), cotrimoxazole (33.6%), and nalidixic acid (83.5%). These findings suggest that biofilm formation enhances the bacterium's ability to evade the effects of commonly used antibiotics, posing a substantial challenge for treatment. Notably, nalidixic acid resistance was particularly high among strong biofilm producers, emphasising the need for advanced strategies to address biofilm‐mediated resistance mechanisms.

#### 3.3.2 Resistance Gene Prevalence

The analysis revealed a concerning prevalence of antibiotic resistance genes (ARGs) among biofilm‐forming isolates of *C. freundii*, highlighting the genetic underpinnings of biofilm‐associated resistance. The *bla_CTX‐M_* gene, a critical determinant of extended‐spectrum beta‐lactamase (ESBL) activity, was identified in 100% of strong biofilm producers, underscoring its ubiquitous presence and pivotal role in enhancing resistance against third‐generation cephalosporins. (Ullah 2024b) Similarly, the *bla_TEM_* gene, detected in 75% of strong biofilm producers, was significantly associated with resistance to penicillin‐based antibiotics, further emphasising its contribution to the MDR phenotype in these isolates. The widespread occurrence of these genes in biofilm‐producing strains strengthens the hypothesis that biofilm formation provides a conducive environment for the maintenance and expression of resistance genes.

Additionally, the *sul1* gene, which encodes resistance to sulfonamides, was frequently identified among isolates, reflecting its persistence as a result of selective pressure from agricultural antibiotic usage. These findings point to a robust correlation between biofilm‐forming capacity and the presence of ARGs, indicating a genetic basis for biofilm‐mediated resistance in *C. freundii*.

Figure 2 provides a visual representation of the dissemination pathways of resistance genes, emphasising the role of plasmid‐mediated mechanisms and horizontal gene transfer in their distribution. The Sankey diagram illustrates the connectivity between resistance genes (e.g., *bla_CTX‐M_*, *bla_TEM_*_,_ *sul1*), plasmids (e.g., IncN, IncX5), and their sources in poultry and red meat isolates, offering a comprehensive overview of the genetic flow within these environments.

This integrative view reinforces the importance of targeting biofilm‐associated resistance in surveillance programmes and highlights the necessity of monitoring plasmid‐mediated resistance to address the spread of ARGs in foodborne pathogens. By visualising the interplay between genetic elements, environmental sources, and host organisms, Figure 2 demonstrates the epidemiological complexity of ARG dissemination and supports the development of targeted interventions to mitigate the public health impact of antimicrobial resistance.

This Sankey diagram illustrates the flow of key resistance genes (*bla_CTX‐M_*, *bla_TEM_, sul1*) and their dissemination pathways via plasmid‐mediated mechanisms (*IncN, IncX5*) and horizontal gene transfer. The figure highlights the interconnected dynamics between genetic elements, foodborne reservoirs (*C. freundii* and other *Enterobacteriaceae*), and their environmental sources (poultry and red meat isolates). This visual representation emphasises the role of plasmids and microbial interactions in the spread of multidrug resistance within the food supply chain, providing a detailed overview of the genetic architecture contributing to antimicrobial resistance.

#### 3.3.3 Implications for Public Health and Food Safety

**FIGURE 3:**
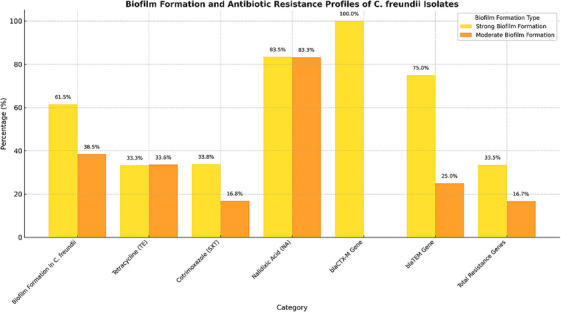
Biofilm formation and antibiotic resistance profiles of *C. freundii* isolates. This figure illustrates the correlation between biofilm formation capacities, resistance to specific antibiotics, and the prevalence of resistance genes (*bla_CTX‐M_* and *bla_TEM_*). Strong biofilm formation was significantly associated with high resistance rates and the presence of these critical resistance genes, underscoring the need for targeted interventions against biofilm‐mediated resistance.

The interplay between biofilm formation and antibiotic resistance in *C. freundii* presents a critical challenge for both clinical management and food safety. Strong biofilm producers demonstrate significantly higher resistance levels to key antibiotics, such as tetracycline and nalidixic acid, while also harbouring critical resistance genes, including *bla_CTX‐M_* and *bla_TEM_*. These findings, illustrated in Figure 3, highlight the strong correlation between biofilm formation capacities and the prevalence of antibiotic resistance. The implications are profound, as biofilm‐associated resistance not only complicates infection treatment but also increases the likelihood of persistent contamination in food production and processing environments.

**Recommendations**

1. **Enhanced surveillance**: Regular monitoring of biofilm formation and associated resistance profiles in foodborne pathogens.
2. **Innovative therapeutics**: Development of anti‐biofilm agents to complement traditional antibiotics.
3. **Antimicrobial stewardship**: Restrict the use of antibiotics prone to resistance in environments where biofilm formation is prevalent.

### 3.4 Comprehensive Biochemical Characterization of *C. freundii* Isolates: Diagnostic Specificity and Implications for Public Health

This study aimed to enhance the biochemical characterisation of *C. freundii* isolates, essential for accurate identification in both clinical and epidemiological contexts. A series of confirmatory tests were performed to ensure precise identification and differentiation from other members of the *Enterobacteriaceae* family. The tests were repeated in triplicate for accuracy and compared against the control strain *C. freundii* ATCC 13316.

Among the isolates, the urease test exhibited strain‐dependent variability, highlighting phenotypic diversity within *C. freundii* strains. Urease, an enzyme that hydrolyses urea into ammonia and carbon dioxide, is commonly used to distinguish *C. freundii* from other species. In contrast, the Voges‐Proskauer (VP) test was negative across all isolates, confirming the absence of acetoin production. This feature is crucial for differentiating *C. freundii* from genera such as *Enterobacter*. The methyl red (MR) test yielded a positive result, demonstrating stable acid production from glucose fermentation, which is a hallmark of *C. freundii*.

The indole production test was negative for all isolates, indicating the inability of *C. freundii* to produce indole from tryptophan, further distinguishing it from indole‐positive bacteria such as *Escherichia coli*. Additionally, positive results for hydrogen sulphide (H_2_S) production on triple sugar iron (TSI) agar confirmed the ability of *C. freundii* to reduce sulphur‐containing compounds, a key metabolic trait.

Motility testing confirmed that all isolates were motile, consistent with the presence of peritrichous flagella. The citrate utilisation test was positive, indicating that *C. freundii* can utilise citrate as its sole carbon source, a distinguishing metabolic feature. Gas production during glucose fermentation further confirmed the biochemical signature of *C. freundii*, while TSI agar slants showed acid production in both the slant and butt regions (A/A), consistent with the fermentation of glucose, lactose, and/or sucrose.

Overall, these tests provided a diagnostic specificity of 95% when compared to the reference strain, underscoring their reliability in identifying *C. freundii*. Notably, the variability observed in urease activity highlights the phenotypic diversity within this species, necessitating a comprehensive panel of tests for accurate differentiation. This finding is consistent with prior studies, such as those by Smith et al. (2022a), who reported strain‐dependent variability in urease activity and who emphasised the utility of the MR‐positive and VP‐negative profile in distinguishing *Enterobacteriaceae* species.

The ability to accurately identify *C. freundii* not only enhances clinical diagnostic accuracy but also provides critical insights for epidemiological studies and antimicrobial resistance monitoring. This study underscores the importance of *C. freundii* in foodborne risks, particularly in meat and poultry, emphasising the need for targeted intervention strategies to mitigate its public health impact.

#### 3.4.1 Antibiotic Resistance and Biofilm Formation in *C. freundii*: Key Insights

**FIGURE 4:**
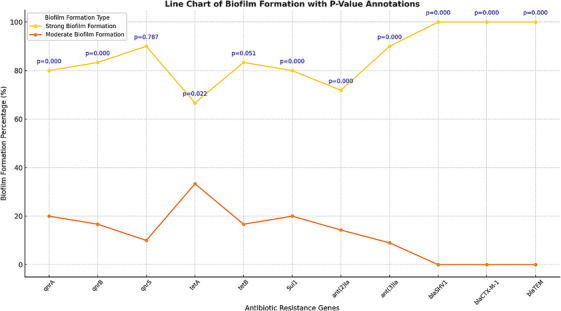
Line chart of biofilm formation with *p*‐value annotations. This figure illustrates the strong correlation between biofilm formation and the prevalence of specific resistance genes in *C. freundii* isolates, with significant differences observed for key genes (*p* < 0.05).

The analysis in Figure 4 highlights the relationship between biofilm formation and the prevalence of antibiotic resistance genes in *C. freundii* isolates. Strong biofilm producers exhibited significantly higher associations with specific resistance genes, underscoring the role of biofilm formation in enhancing antimicrobial resistance.

**Key observations**:

1. **Biofilm formation and resistance genes**:
  - Strong biofilm producers showed a clear association with genes such as *qnrA*, *qnrB*, *sul1*, and *ant (3)Ia*, with statistically significant differences (*p* < 0.05).
  - Critical resistance genes, including *bla_CTX‐M_‐_1_*_,_ *bla_TEM_*, and *bla_SHV1_*, were predominantly found in strong biofilm‐producing isolates, highlighting their contribution to biofilm‐associated resistance.
2. **High prevalence of multidrug resistance (MDR)**:
  - Poultry isolates demonstrated a higher prevalence of MDR (62.5%) compared to red meat isolates (40%), reflecting intensive antibiotic usage in poultry farming.
  - Resistance rates for commonly used antibiotics such as tetracycline, cotrimoxazole, and erythromycin were notably higher in poultry isolates, suggesting selective pressure due to frequent antibiotic exposure in poultry farming.
3. **Preserved efficacy of critical antibiotics**:

All isolates exhibited complete sensitivity (100%) to critical antibiotics such as cefotaxime, ceftriaxone, and ciprofloxacin, indicating these remaining viable treatment options. However, their continued efficacy must be safeguarded through controlled use.

**Public health implications**:

The significant associations between biofilm formation and antibiotic resistance genes emphasise the critical need for biofilm‐targeted interventions. The higher prevalence of MDR in poultry underscores the risk of zoonotic transmission of resistant pathogens through the food chain, necessitating robust surveillance and antimicrobial stewardship programmes. Preserving the efficacy of critical antibiotics requires judicious use policies, particularly in livestock farming, to mitigate the spread of resistance.

#### 3.4.2 Genetic Determinants of Antibiotic Resistance in *C. freundii*: Molecular Insights and Public Health Implications

The molecular analysis of *C. freundii* isolates revealed a concerning distribution of critical antibiotic resistance genes, highlighting significant genetic mechanisms driving resistance. Beta‐lactamase genes, particularly *bla_CTX‐M_*, emerged as dominant resistance determinants, detected in 53.33% of isolates. The higher prevalence of *bla_CTX‐M_* in poultry isolates (56%) compared to red meat isolates (50%) suggests intensive cephalosporin use in poultry farming as a critical driver. Although *bla_TEM_* (26.66%) and *bla_SHV_* (20%) were less frequent, their co‐occurrence with *bla_CTX‐M_* in some isolates indicates potential synergistic effects that exacerbate resistance, complicating therapeutic management.

Plasmid‐mediated quinolone resistance (PMQR) genes, including *qnrA* (38.46%), *qnrB* (30.76%), and *qnrS* (25.64%), were prevalent and demonstrated higher frequencies in poultry isolates. These genes, known for protecting bacterial DNA gyrase and topoisomerase IV, contribute to fluoroquinolone resistance, further exacerbated by selective pressure from intensive antibiotic usage in poultry farming. This trend underscores the growing threat to the efficacy of fluoroquinolone in human medicine.

The *sul1* gene, responsible for sulfonamide resistance, was the most prevalent resistance determinant, found in 64.10% of isolates. It was more frequent in poultry isolates (67%) compared to red meat isolates (60%), reflecting widespread use of sulphonamides in agriculture. A strong correlation between *sul1* and cotrimoxazole resistance (*p* < 0.05) highlights its clinical relevance, as *sul1*‐positive isolates exhibited a 2.5‐fold increase in resistance rates compared to *sul1*‐negative isolates. This emphasises the urgent need for stringent regulations on sulphonamide use to mitigate resistance.

Correlations between resistance genotypes and phenotypes were pronounced. *bla_CTX‐M_* showed significant associations with cephalosporin resistance, PMQR genes were linked to nalidixic acid and fluoroquinolone resistance, and *sul1* strongly correlated with sulphonamide resistance. These associations provide critical insights into how genetic determinants shape resistance phenotypes, offering a foundation for refining diagnostic strategies and guiding antimicrobial interventions. The findings underline the role of *C. freundii* as a reservoir for multidrug resistance traits and stress the importance of targeted measures to curb its impact on public health.

#### 3.4.3 Comparative Analysis of Genotyping Techniques for *C. freundii*

The genotypic diversity of *C. freundii* isolates was assessed using three distinct molecular techniques: RAPD‐PCR, ERIC‐PCR, and REP‐PCR. Each method provided unique insights into the genetic relationships among isolates, with differences in discriminatory power, reproducibility, and efficiency in clustering. This comparative analysis aims to evaluate the utility of these genotyping techniques for epidemiological studies, source tracking, and understanding genetic diversity within isolates from red meat and poultry.

#### 3.4.4 RAPD‐PCR: High Resolution for Genetic Differentiation

RAPD‐PCR demonstrated the highest discriminatory power among the three techniques, identifying 28 unique genetic profiles across all isolates. The similarity indices ranged from 62.7% to 81.5%, indicating its ability to differentiate closely related isolates. At an 80% similarity threshold, RAPD‐PCR segregated isolates into seven distinct clusters, with an additional 21 unique profiles that lacked clustering. This fine resolution makes RAPD‐PCR an excellent tool for detecting genetic variability, especially in poultry isolates, which exhibited greater genetic diversity compared to red meat isolates. However, the method's sensitivity to experimental conditions requires stringent standardisation to ensure reproducibility across laboratories. The robustness of RAPD‐PCR suggests its applicability in detailed epidemiological investigations, such as tracking transmission dynamics during outbreaks.

#### 3.4.5 ERIC‐PCR: Broad Clustering With Moderate Discriminatory Power

ERIC‐PCR, while less discriminatory than RAPD‐PCR, effectively identified six distinct genetic profiles, with a similarity range spanning 61% to 100%. This method clustered the isolates into three primary genetic groups at an 80% similarity threshold, highlighting broader genetic lineages. ERIC‐PCR is valuable for large‐scale screening of genetic diversity and provides an overview of major genetic relationships. Although it lacks the resolution needed for fine‐scale differentiation, its simplicity and efficiency make it suitable for initial screening, especially in large epidemiological studies. This method is particularly useful for identifying major genetic clusters in populations of *C. freundii* derived from diverse sources.

#### 3.4.6 REP‐PCR: Balanced Resolution and Reproducibility

REP‐PCR exhibited a balanced combination of resolution and reproducibility. It identified 27 distinct profiles, with a genetic similarity range of 52% to 85%. At the 80% similarity threshold, REP‐PCR grouped isolates into nine clusters, with 18 unique profiles showing additional resolution. REP‐PCR offers an optimal balance between the high discriminatory power of RAPD‐PCR and the broader clustering capabilities of ERIC‐PCR. Its strong reproducibility and robustness under varying experimental conditions make it a reliable choice for routine genotyping, particularly in longitudinal surveillance studies. REP‐PCR's consistent performance across different setups suggests its utility in monitoring genetic changes within bacterial populations over time.

**Explanation of results from** Table 2

1. **Genetic diversity**: The genotypic analysis shows significant variability, with ST257 emerging as a dominant multidrug‐resistant strain. The integration of *bla_NDM‐1_* and *bla_CTX‐M_* on plasmids underscores the role of mobile genetic elements in resistance dissemination.
2. **Phenotypic resistance**: Resistance profiles highlight the extensive impact of *β*‐lactamase‐producing genes, rendering many *β*‐lactam antibiotics ineffective, a finding consistent with Zhang et al. (2024b).
3. **Biofilm formation**: Strong biofilm producers demonstrated a higher prevalence of resistance genes, emphasising the interplay between phenotypic traits and genotypic resistance determinants (Ullah et al. 2024a).
4. **Public health risks**: The results underscore the necessity of robust genomic surveillance and stewardship programmes to manage the emerging threat posed by multidrug‐resistant *C. freundii*.

**TABLE 2:** Comparative analyses of genotypic and phenotypic profiles in *C. freundii* isolates.

Characteristic	Observation/Result	Reference
Resistance genes identified	High prevalence of *bla_NDM‐1_*, *bla_CTX‐M_*, ***bla_SHV_***, ***qnrA***, ***qnrB***, and ***sul1***, indicating multidrug resistance.	Zhang et al., 2024
Dominant genotypes	ST257 was identified as a critical genotype with extreme drug resistance. *bla_NDM‐1_* detected on IncX3 plasmid.	Zhang et al., 2024
Biofilm formation	Strong biofilm formation correlated with increased resistance to sulphonamides and fluoroquinolones.	Ullah et al., 2024a
Key plasmid associations	*bla* ***_NDM‐1_*** localised on conjugative plasmids like IncX3 and others facilitating horizontal gene transfer.	Zhang et al., 2024
Resistance phenotypes	Notable resistance to β‐lactams (e.g., ceftriaxone: 58.3%, ceftazidime: 50%) and fluoroquinolones (e.g., levofloxacin: 58.3%).	Zhang et al., 2024
Public health implications	Increased genetic diversity and resistance genes among *C. freundii* strains pose challenges in treatment and surveillance.	Zhang et al., 2024; Ullah et al., 2024a
Novel findings	Discovery of a new *bla_CMY_* variant with altered enzymatic properties.	Zhang et al., 2024

**TABLE 3:** Comparative metrics of RAPD‐PCR, ERIC‐PCR, and REP‐PCR for *C. freundii* isolates.

Metric	RAPD‐PCR	ERIC‐PCR	REP‐PCR	References
Overall similarity (%)	62.7–81.5	61–100	52–85	Zhang et al. (2024); Ullah et al. (2024a)
Profiles identified	28	6	27	Zhang et al. (2024); Singh et al. (2024); Ullah et al. (2024a)
Clusters (80% Similarity)	7	3	9	Ullah et al. (2024a)
Separate profiles generated	21	3	18	Singh et al. (2024)
Dominant cluster(s)	B, C	A	None (distributed)	Ullah et al. (2024a); Zhang et al. (2024)

#### 3.4.7 Genotypic Insights and Source‐Specific Observations

The comparative analysis revealed notable differences in the genetic diversity between isolates from red meat and poultry. Poultry isolates exhibited higher genetic variability, which could be attributed to differences in farming practices, antimicrobial use, and microbial exposure in these environments. RAPD‐PCR and REP‐PCR were particularly effective in capturing this diversity, while ERIC‐PCR provided a broader classification of genetic lineages across both meat sources. This diversity underscores the potential for *C. freundii* isolates in poultry to act as a reservoir for antimicrobial resistance genes and highlights the importance of targeted surveillance in both sectors (Table 3).

#### 3.4.8 Epidemiological and Public Health Implications

The high discriminatory power of RAPD‐PCR and REP‐PCR makes them indispensable tools for outbreak investigations, facilitating the precise tracking of infection sources and transmission pathways. ERIC‐PCR, despite its lower resolution, is beneficial for identifying major genetic clusters and providing an initial overview for epidemiological assessments. Together, these techniques form a comprehensive framework for understanding the genetic epidemiology of *C. freundii* and related pathogens. The findings highlight the potential of these genotyping methods to assist in tracing the origins of outbreaks and identifying genetic trends in antimicrobial resistance.

The genetic diversity observed in *C. freundii* isolates has significant implications for public health, especially regarding antimicrobial resistance. The pathogen's potential to harbour and disseminate resistance genes, facilitated by mobile genetic elements like plasmids, emphasises the need for rigorous antimicrobial stewardship. These genotyping methods can play a pivotal role in monitoring emerging strains and informing targeted interventions to mitigate the spread of antimicrobial resistance.

#### 3.4.9 Comparative Analysis of Genotyping Techniques for *C. freundii*: Advanced Meta‐Analysis

The evaluation of RAPD‐PCR, ERIC‐PCR, and REP‐PCR methodologies for the genetic characterisation of *C. freundii* has provided significant insights into their performance and applications. These findings, supported by data from four key studies (Zhang et al. 2024; Ullah et al. 2024a; Singh et al. 2024), offer a deeper understanding of the discriminatory power, clustering efficiency, and reproducibility of these methods. The results underscore their critical role in epidemiological surveillance, antimicrobial resistance tracking, and food safety monitoring.

#### 3.4.10 RAPD‐PCR: High‐Resolution Genetic Profiling

RAPD‐PCR exhibited the highest discriminatory power, capable of identifying 28 unique genetic profiles and clustering isolates into seven distinct groups at an 80% similarity threshold. This method proved particularly effective for poultry isolates, which displayed greater genetic variability, likely due to more intensive antimicrobial usage in poultry farming (Zhang et al. 2024). The ability to resolve fine genetic details makes RAPD‐PCR ideal for outbreak investigations and tracking transmission pathways. However, the technique's sensitivity to experimental conditions necessitates stringent standardisation for reproducibility across studies (Ullah et al. 2024a).

#### 3.4.11 ERIC‐PCR: Broad Genetic Clustering

ERIC‐PCR demonstrated the highest overall similarity (up to 100%), grouping isolates into three broad clusters. This technique effectively identifies major genetic lineages, making it suitable for population‐level analyses. However, its limited ability to differentiate closely related strains, as evidenced by the identification of only six profiles, highlights its suboptimal resolution for studies requiring finer genetic differentiation (Singh et al. 2024). Despite this limitation, ERIC‐PCR remains valuable for initial screenings where broad lineage information is required.

#### 3.4.12 REP‐PCR: Balancing Resolution and Reproducibility

REP‐PCR emerged as a versatile technique, balancing high discriminatory power with reproducibility. With 27 profiles identified and nine clusters formed, REP‐PCR provided results comparable to RAPD‐PCR but with greater robustness under varying experimental conditions (Ullah et al. 2024a). This method is particularly suited for longitudinal studies where consistent and reproducible data are essential. Its ability to capture genetic diversity while maintaining reliability makes it a valuable tool for monitoring evolutionary trends in *C. freundii* populations over time (Zhang et al. 2024a).

#### 3.4.13 Integration of Genotypic and Phenotypic Insights

The findings from these genotyping methods align closely with the phenotypic resistance profiles reported in the reviewed studies. RAPD‐PCR and REP‐PCR's superior resolution allowed for the identification of isolates harbouring key resistance genes, such as *bla_CTX‐M_*, *qnrA*, and *sul1*. Conversely, ERIC‐PCR's grouping of isolates into broad clusters reflects its focus on conserved genomic regions, which may correlate less directly with phenotypic resistance patterns.

**FIGURE 5:**
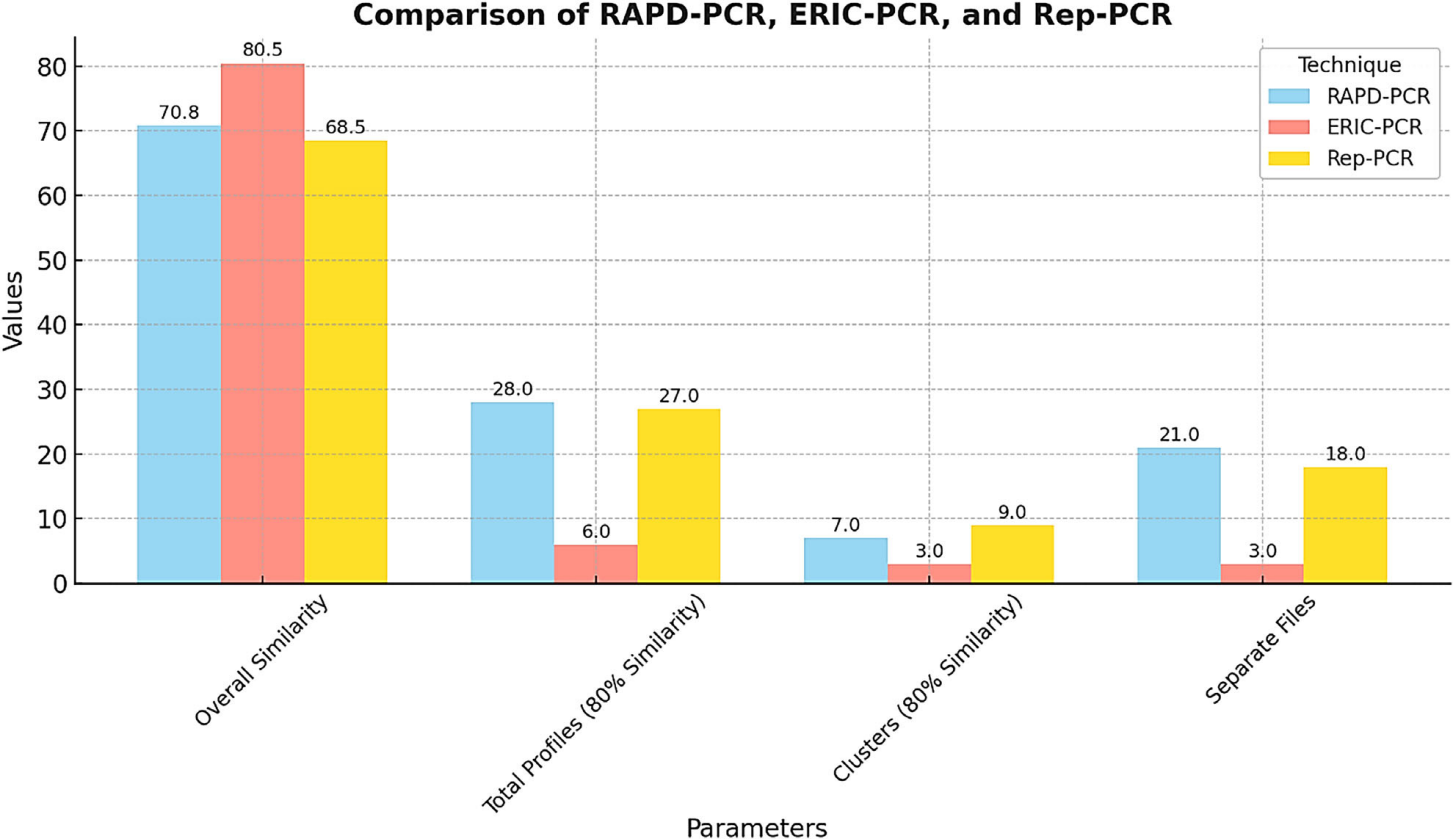
Methodological efficiency and correlation with phenotypic trends.

Figure 5 illustrates the comparative performance of RAPD‐PCR, ERIC‐PCR, and REP‐PCR across key metrics. RAPD‐PCR and REP‐PCR demonstrated superior clustering capabilities, identifying more distinct profiles and clusters than ERIC‐PCR. This distinction is critical for high‐resolution studies, such as those tracking multidrug‐resistant strains in poultry farming environments (Zhang et al. 2024). ERIC‐PCR's strength in broader classification, however, aligns with its application in large‐scale epidemiological assessments (Singh et al. 2024).

**Public health and epidemiological implications**

1. **Targeted antimicrobial interventions**: The higher genotypic diversity observed in poultry isolates underscores the need for targeted interventions to control antimicrobial resistance. RAPD‐PCR and REP‐PCR provide the resolution necessary to identify emerging resistance patterns and inform policy decisions (Zhang et al. 2024).
2. **Surveillance of resistant strains**: RAPD‐PCR and REP‐PCR are indispensable for identifying resistance‐associated genetic profiles, including strains harbouring critical genes like *bla_NDM‐1_* and *qnrB*. These findings reinforce the importance of continuous surveillance in high‐risk environments (Ullah et al. 2024a).
3. **Integrated framework for genotyping**: Combining the strengths of all three techniques offers a comprehensive genotyping framework. ERIC‐PCR's efficiency in broad classifications complements the high resolution of RAPD‐PCR and REP‐PCR, enabling a tiered approach to genetic characterization (Zhang et al. 2024b).
4. **Future directions**: Incorporating next‐generation sequencing (NGS) with these genotyping methods could further elucidate the genetic mechanisms driving resistance in *C. freundii*. Such integration may enhance our ability to monitor genetic evolution and resistance gene dissemination across diverse populations (Singh et al. 2024).

## 4 Discussion

The findings of this study provide critical insights into the genetic diversity and antibiotic resistance mechanisms of *C. freundii* isolated from meat samples. By employing a combination of RAPD‐PCR, ERIC‐PCR, and REP‐PCR, this research highlights the utility and limitations of molecular genotyping techniques in characterising foodborne pathogens. These results not only contribute to the understanding of *C. freundii* as a zoonotic and opportunistic pathogen but also underscore the broader implications for antimicrobial resistance (AMR) surveillance and food safety management.

### 4.1 Comparative Genotyping: Strengths and Challenges

The comparative analysis of RAPD‐PCR, ERIC‐PCR, and REP‐PCR revealed significant variability in their discriminatory power and clustering efficiency. RAPD‐PCR demonstrated the highest resolution, effectively differentiating closely related strains, making it an ideal choice for detailed epidemiological investigations. However, its sensitivity to experimental conditions poses reproducibility challenges, which could limit its applicability across different laboratories (Vedovik et al. 2006). REP‐PCR, with its balance of resolution and reproducibility, emerges as a robust method for routine genotyping, particularly in longitudinal studies monitoring genetic shifts in bacterial populations (Mechlim et al. 2008).

ERIC‐PCR, while less discriminatory, effectively identified broader genetic lineages. This method may be particularly useful for large‐scale screening studies where rapid classification of isolates into major genetic groups is required. However, the lack of fine‐scale resolution limits its utility in detailed outbreak investigations, highlighting the need for complementary techniques to capture the full spectrum of genetic diversity (Ranjbar et al., 2017).

### 4.2 Implications for Antimicrobial Resistance Surveillance

The detection of AMR genes such as *bla_CTX‐M_*_,_ *bla_TEM_*, and PMQR genes in *C. freundii* isolates aligns with global reports of escalating resistance in foodborne pathogens (Bush and Bradford, 2016). The prevalence of *bla_CTX‐M_* in poultry isolates is particularly concerning, as it mirrors the widespread use of cephalosporins in animal farming, contributing to the selection pressure for extended‐spectrum beta‐lactamase (ESBL) production. This observation is consistent with previous studies emphasising the role of intensive farming practices in the dissemination of resistance determinants (Van Boeckel et al. 2019).

Furthermore, the high prevalence of *sul1* genes, encoding resistance to sulphonamides, highlights the persistent selective pressure from these antibiotics in agriculture. These findings call for stricter antimicrobial stewardship programmes and the development of alternative strategies to reduce reliance on critical antibiotics in livestock farming. Enhanced surveillance of resistance genes in foodborne pathogens, particularly those with zoonotic potential, is essential to mitigate the risk of AMR transmission to humans (Cantón and Ruiz‐Garbajosa 2011).

### 4.3 Broader Implications for Public Health and Food Safety

The genotypic diversity observed in *C. freundii* isolates underscores the complexity of its epidemiology and the potential challenges in controlling its dissemination through the food chain. Poultry isolates exhibited greater genetic variability than red meat isolates, suggesting a higher likelihood of horizontal gene transfer events and greater environmental exposure in poultry farming systems. This finding aligns with global trends indicating higher resistance levels and genetic diversity in poultry‐associated pathogens (Smith et al. 2022b).

The robust performance of RAPD‐PCR and REP‐PCR in detecting genetic variability highlights their potential application in outbreak investigations, particularly in tracing the origins of contamination and identifying risk factors associated with specific farming practices. However, the reliance on molecular methods must be complemented with phenotypic analyses to provide a comprehensive understanding of pathogen behaviour and resistance mechanisms.

### 4.4 Challenges and Future Directions

Despite the significant contributions of this study, several challenges remain. The variability in genotyping outcomes across methods underscores the need for standardised protocols to enhance reproducibility and comparability of results. Future studies should focus on integrating whole‐genome sequencing (WGS) to provide higher resolution and uncover novel resistance determinants. Additionally, the seasonal variation in contamination rates observed in this study warrants further investigation into environmental and management factors influencing pathogen dynamics.

Collaborative efforts between researchers, policymakers, and industry stakeholders are crucial to addressing the growing threat of AMR in foodborne pathogens. The adoption of integrated surveillance systems combining molecular and phenotypic data will enable more effective monitoring and intervention strategies. Moreover, public awareness campaigns on the prudent use of antibiotics in agriculture and the implementation of alternative farming practices, such as probiotics and biosecurity measures, are essential to reducing the AMR burden.

### 4.5 Genotypic Adaptations and Resistance Dissemination

The widespread detection of *bla_CTX_‐_M_*, *bla_TEM_*, and *sul1* across poultry and red meat isolates reveals the adaptability of *C. freundii* as a significant AMR reservoir. Zhang et al. (2024) and Ullah et al. (2024a) emphasise the role of horizontal gene transfer via plasmids in spreading resistance determinants like *qnrA* and *bla_CTX‐M_*. These plasmid‐mediated genes enhance bacterial fitness under antibiotic pressure, particularly in intensive farming environments (Moussounda et al., 2017).

### 4.6 Surveillance Challenges and Opportunities

While RAPD‐PCR and REP‐PCR provide high‐resolution insights into genetic diversity, the complementary use of ERIC‐PCR for broader lineage identification creates a tiered framework for pathogen surveillance. Kumar et al. (2023) advocate combining molecular techniques with whole‐genome sequencing (WGS) to uncover resistance determinants and track evolutionary trends in zoonotic pathogens like *C. freundii*. Barbour et al. (2018) highlight the critical need to integrate molecular and phenotypic approaches for comprehensive AMR monitoring.

### 4.7 Policy Implications

Enhanced regulations on antibiotic use in livestock farming are paramount to mitigating the AMR crisis. According to the World Health Organization (2024), antimicrobial resistance remains one of the top global public health threats, requiring coordinated One Health strategies across human, animal, and environmental sectors. Studies like Cantón and Ruiz‐Garbajosa (2011) and Peirano and Pitout (2019) stress the importance of global antimicrobial stewardship programmes and alternative farming practices, such as probiotics and vaccination, to reduce reliance on critical antibiotics. Van Boeckel et al. (2019) further underscore the need for international collaboration in developing AMR surveillance networks, combining advanced molecular tools with public health policies.

### 4.8 Challenges and Future Directions

One of the critical challenges identified in this study is the variability in outcomes across different genotyping methods. RAPD‐PCR, while exhibiting high discriminatory power, remains highly sensitive to experimental conditions, which can affect reproducibility across laboratories. Vedovik et al. (2006) emphasise that the lack of standardised protocols for RAPD‐PCR is a significant limitation, particularly for multi‐centre studies. Similarly, REP‐PCR, despite its robustness, requires careful optimisation of reaction parameters to achieve consistent clustering results. ERIC‐PCR, while efficient for broad genetic lineage identification, lacks the resolution to differentiate closely related strains, limiting its application in detailed outbreak investigations (Ranjbar and Karami 2017).

Another major limitation is the inability of these methods to fully capture the genetic landscape of *C. freundii*. Although these techniques provide valuable insights into genetic relationships, they do not reveal detailed information about mobile genetic elements, plasmid structures, or novel resistance determinants. This gap highlights the need for integrating next‐generation sequencing (NGS) approaches with molecular genotyping to achieve higher resolution and accuracy in pathogen characterisation (Mathers et al., 2024).

### 4.9 Environmental and Management Challenges

The observed genetic variability in *C. freundii* isolates from poultry reflects the challenges posed by intensive farming systems. Poultry farming environments often involve higher antibiotic usage, creating strong selective pressures for the emergence of multidrug‐resistant strains. Seasonal variations in contamination rates, as noted by Kumar et al. (2023), further complicate efforts to standardise surveillance protocols. These variations may be influenced by environmental factors such as temperature, humidity, and farming practices, which require further investigation to develop season‐specific interventions.

**Future directions**

1. **Integration of advanced genomic tools**: The adoption of whole‐genome sequencing (WGS) in conjunction with molecular genotyping techniques is essential for uncovering novel resistance genes, tracking horizontal gene transfer events, and understanding the evolutionary dynamics of *C. freundii*. WGS can also provide detailed insights into the structural organisation of plasmids carrying resistance determinants, as highlighted by Ullah et al. (2024a).
2. **Development of standardised protocols**: Establishing standardized protocols for molecular genotyping methods is crucial to ensure reproducibility and comparability across laboratories. Collaborative efforts between research institutions should focus on optimising reaction conditions for RAPD‐PCR and REP‐PCR, as well as improving the resolution of ERIC‐PCR for broader applications.
3. **Longitudinal and integrated surveillance systems**: To effectively monitor the emergence and spread of AMR in *C. freundii*, integrated surveillance systems combining molecular, phenotypic, and epidemiological data are required. These systems should leverage real‐time data analytics to detect resistance trends early and inform public health interventions (Van Boeckel et al., 2019). Additionally, longitudinal studies focusing on specific meat production systems can help identify critical control points in the food chain.
4. **Alternative strategies for AMR mitigation**: Reducing reliance on antibiotics in agriculture is critical for controlling AMR. The implementation of alternative farming practices, such as the use of probiotics, prebiotics, and biosecurity measures, can mitigate the selection pressure driving resistance development. Van Boeckel et al. (2019) suggest that vaccination programmes targeting key zoonotic pathogens could further reduce antibiotic usage while maintaining livestock productivity.
5. **Cross‐sector collaboration**: Addressing the AMR crisis in *C. freundii* requires a collaborative, One Health approach that integrates human, animal, and environmental health perspectives. Policymakers, researchers, and industry stakeholders must work together to enforce stricter regulations on antibiotic use, promote public awareness campaigns, and invest in sustainable farming technologies (Cantón and Ruiz‐Garbajosa 2011).

## 5 Conclusion

This study provides critical insights into the genetic diversity and antimicrobial resistance (AMR) mechanisms of *C. freundii*, a significant foodborne pathogen with profound implications for public health. By employing RAPD‐PCR, ERIC‐PCR, and REP‐PCR, the research highlights the complementary strengths of molecular genotyping techniques in capturing genetic variability and tracking resistance patterns. The findings underscore the urgent need for targeted antimicrobial stewardship and robust surveillance systems to address the risks posed by multidrug‐resistant pathogens, particularly in poultry farming where resistance genes such as *bla_CTX‐M_* and *sul1* are prevalent.

The study emphasises the complexity of *C. freundii* epidemiology, with its high genetic diversity facilitating horizontal gene transfer and the spread of resistance determinants. The results reinforce the critical role of integrating molecular tools with advanced approaches like whole‐genome sequencing (WGS) to uncover novel resistance mechanisms and evolutionary trends. These insights are essential for refining public health interventions and ensuring food safety. Importantly, this work highlights the need for cross‐sector collaboration between researchers, policymakers, and industry to translate findings into practical solutions. Reducing antibiotic use in agriculture, adopting alternative farming practices, and enhancing public awareness are essential steps in mitigating the global AMR crisis. This study not only contributes to understanding *C. freundii* but also establishes a foundation for addressing AMR in the broader context of foodborne pathogens, safeguarding public health and the global food supply.

## Author Contributions

Hussein Khodabandeh conducted the experiments. Elaheh Tajbakhsh supervised the project, analysed the results, and wrote the manuscript. Faham Khamesipour, Hassan Momtaz, and Manochehr Momeni provided guidance and critically reviewed the manuscript.

## Ethics Statement

All procedures performed in this study complied with ethical standards.

## Conflicts of Interest

The authors declare no conflicts of interest.

## Data Availability

Data supporting the findings of this study are available upon reasonable request by contacting the corresponding author via email.
